# Hospitalizations due to respiratory syncytial virus (RSV) infections in Germany: a nationwide clinical and direct cost data analysis (2010–2019)

**DOI:** 10.1007/s15010-023-02122-8

**Published:** 2023-11-16

**Authors:** Patricia Niekler, David Goettler, Johannes G. Liese, Andrea Streng

**Affiliations:** Department of Pediatrics, University Hospital of Würzburg, Josef-Schneider-Str. 2, 97080 Würzburg, Germany

**Keywords:** Respiratory syncytial virus, Healthcare costs, Hospitalization, ICD-10, Epidemiology, Germany

## Abstract

**Purpose:**

Clinical and direct medical cost data on RSV-related hospitalizations are relevant for public health decision-making. We analyzed nationwide data on RSV-coded hospitalizations from Germany in different age and risk groups.

**Methods:**

Assessment of RSV-coded hospitalizations (ICD-10-GM RSV-code J12.1/J20.5/J21.0 as primary discharge diagnosis) from 01/2010 to 12/2019, using remote data retrieval from the Hospital Statistics Database of the German Federal Statistical Office.

**Results:**

Overall, 205,352 RSV-coded hospitalizations (198,139 children < 18 years, 1,313 adults, 5,900 seniors > 59 years) were reported (median age < 1 year, IQR 0; 1; 56% males, 32% with RSV pneumonia). Annual median RSV-coded hospitalization incidence was 24.8/100,000 persons (IQR 21.3; 27.5); children reported a median incidence of 145.8 (IQR 130.9; 168.3). Between 2010 and 2019, hospitalization incidence increased 1.7-fold/15.1-fold/103-fold in children/adults/seniors. Adults and seniors reported higher rates of underlying chronic conditions, complications, and intensive care treatment than children; of 612 in-hospital fatalities, 103/51/458 occurred in children/adults/seniors. Per-patient mean costs varied between 3286€ ± 4594 in 1–4-year-olds and 7215€ ± 13,564 among adults. Increased costs were associated with immune disorders (2.55-fold increase compared to those without), nervous system disorders (2.66-fold), sepsis (7.27-fold), ARDS (12.85-fold), intensive care (4.60-fold) and ECMO treatment (16.88-fold).

**Conclusion:**

The economic burden of RSV-related hospitalizations in Germany is substantial, even when only considering cases with RSV-coded as the primary discharge diagnosis. Children represented the vast majority of RSV-coded hospitalizations. However, adults and seniors hospitalized for RSV were at a higher risk of severe complications, required more costly treatments, and had higher fatality rates; although their RSV-coded hospitalization incidence showed a clear upward trend since 2017, their true hospitalization incidence is still likely to be underestimated due to lack of routine RSV testing in these age groups. Hence, new treatments and vaccines for RSV ideally should also target adults and seniors in addition to children.

**Supplementary Information:**

The online version contains supplementary material available at 10.1007/s15010-023-02122-8.

## Background

Respiratory syncytial virus (RSV) is one of the global leading causes of lower respiratory tract infections (LRTI) in children and the leading cause of hospitalization in children < 1 year old [1]. Especially during the first 6 months of life, RSV contributes substantially to morbidity and mortality burdens globally [2]. Severe outcomes, hospitalization and even death can occur in previously healthy children who are infected with RSV [3]. Specific chronic underlying conditions such as pre-term birth, cardiac malformation, or chronic lung disease are known risk factors for a severe course of pediatric RSV disease [4]. Frequent complications are bronchiolitis, bronchitis and pneumonia, and acute otitis media [4, 5]. However, RSV is also responsible for a significant burden of disease among adults and seniors [6]. Although RSV infections in these age groups are generally considered milder, multiple studies have shown that a large proportion of RSV mortality in industrialized countries occurs in senior patients older than 65 years [7–9]. Adults and seniors with RSV LRTI frequently require hospitalization and are more likely to need admission to the intensive care unit and receive mechanical ventilation [10]. In addition, seniors can experience cardiorespiratory sequelae after respiratory infections caused by RSV [11]. These factors lead to higher health care resource utilization and costs [5]. There are currently two approved RSV vaccines in Europe: one only for individuals over the age of 60 and another for pregnant mothers and for individuals over the age of 60 [12, 13]. Currently in Germany, prophylaxis with monoclonal antibodies is recommended for selected pediatric risk groups, and there is still no widely available RSV vaccine or effective causal treatment available for the general population [14]. This is not unique to Germany, however, as symptomatic relief and supportive care are the only clinical management strategies in other countries as well [15]. With additional RSV vaccine candidates and new prophylactic treatment currently in development, relevant, up-to-date disease burden estimates and cost data are especially important. In the present nationwide study, we analyze clinical burden and cost of RSV-coded hospitalizations in different age and risk groups in Germany.

## Methods

### Study design

We conducted a retrospective observational cost-of-illness study of RSV-coded hospitalizations using the German Federal Statistical Office (DeStatis) database on German Hospital Statistics, which is based on mandatory annual reporting of key information from all hospitals in Germany [16]. The background information on this database and the principal study design and method were previously published in a similar investigation on influenza-related hospitalizations [17]. In brief, the database contains patient hospital discharge diagnoses according to the International Classification of Diseases, 10th Editions, German modification (ICD-10-GM), with the primary diagnosis representing the reason for hospital admission and secondary diagnoses representing comorbidities and complications. Furthermore, the database contains Operation and Procedure Codes (OPS) and information on direct medical costs per patient as allocated for hospital re-imbursement based on diagnosis-related groups (DRG) [18–20]. Data from DeStatis are available for academic institutions on reasonable request, by remote data query on fully anonymized patient datasets.

### Case selection

The database was screened for in-patients of all age groups discharged after receiving treatment at a hospital between 01 January 2010 and 31 December 2019 due to RSV as defined by a primary discharge diagnosis of ICD-10-GM code J12.1 (RSV pneumonia), J20.5 (RSV bronchitis), or J21.0 (RSV bronchiolitis). The codes J12.1, J20.5, and J21.0 are usually allocated to patients with a laboratory-confirmed RSV infection, however, virological confirmation is not mandatory. The RSV-code B97.4! is used solely as a secondary diagnosis, for those patients who test positive for RSV but who have a different primary diagnosis—therefore, this code was not included in the initial case selection. Patients hospitalized for less than 24 h are counted as in-patients with a hospital length of stay of one day.

Potential risk factors/underlying chronic conditions and complications reported as secondary ICD-10-GM diagnosis codes as well as relevant treatment codes were pre-selected for data analysis due to their well-established association with RSV (Supplementary Table 1). Additional selected risk factors/underlying chronic conditions and complications were analyzed in the context of certain age groups (e.g., prematurity as a risk factor was only analyzed among the children age groups).

### Study outcome

The clinical burden of diseases associated with RSV-hospitalization was presented by selected variables (Supplementary Table 1) and stratified by age group. Direct medical hospitalization costs as reported in the database were compared among age groups, study year, and pre-specified selected risk factors/underlying chronic conditions, complications, treatments, and outcomes. Costs were standardized in 2019 Euros using the German Consumer Price index [21].

### Statistical analyses

The annual RSV-coded hospitalization incidence rate per 100,000 persons was stratified by three main age groups: children < 18, adults 18–59, and seniors > 59 years. German national census data served as denominator. Using IBM SPSS Statistics Version 29 (IBM Corporation, One New Orchard Road, Armonk, New York, USA), nominal data were reported as number of persons (percent); p-values were calculated using asymptotic Chi-Squared test. Continuous data were reported as mean (standard deviation) and median with interquartile range (IQR); p-values were calculated using Mann–Whitney *U*-test or Kruskal–Wallis *H*-test, as appropriate. Results were considered statistically significant when *p* < 0.05 and highly significant when *p* < 0.001.

## Results

### ICD-10-coded RSV hospitalizations

During January 2010 and December 2019, 205,352 patients (198,139 children < 18 years [96.5%], 1313 adults 18–59 years [0.6%], 5900 seniors > 59 years [2.9%]) were hospitalized across Germany due to an ICD-10-coded RSV infection with a primary discharge diagnosis of J12.1 (31.6%), J20.5 (30.8%), or J21.0 (37.7%). Out of all patients hospitalized, 44.1% were females; 4 of these (< 0.01%) were pregnant (Table 1). Among all patients the overall mean age was 2.9 years old (SD ± 13.7); those < 1 year accounted for 77.2% of all 198,139 pediatric cases, while the second largest group, children aged 1–4 years old, accounted for 21.9% (Supplementary Table 2).

**Table 1:** General characteristics of patients with RSV-coded hospitalization (ICD-10-GM RSV-code J12.1, J20.5 or J21.0 reported as primary diagnosis) in Germany, January 2010–December 2019

	Age, years			
	All	< 18	18–59	> 59
All cases, *N* (%)	205,352 (100)	198,139 (100)	1313 (100)	5900 (100)
Age (years), median (IQR)	0 (0;1)	0 (0;0)	50 (37;56)	79 (72;85)
Age (years), mean (± SD)	2.93 (± 13.67)	0.40 (± 1.05)	45.81 (± 12.13)	78.65 (± 8.96)
Female sex, *N* (%)	90,466 (44.1)	86,546 (43.7)	601 (45.8)	3319 (56.3)
Selected risk factors/underlying chronic conditions, *N* (%)				
Immune disorder	1907 (0.9)	597 (0.3)	469 (35.7)	841 (14.3)
Chronic disease of LRT (without asthma)	3283 (1.6)	1841 (0.9)	232 (17.7)	1210 (20.5)
Bronchial asthma	1614 (0.8)	1341 (0.7)	69 (5.3)	204 (3.5)
Disease of circulatory system	7484 (3.6)	2038 (1.0)	552 (42.0)	4894 (82.9)
Disease of nervous system	4000 (1.9)	2468 (1.2)	245 (18.7)	1287 (21.8)
*Cerebral palsy*	1014 (0.5)	716 (0.4)	62 (4.7)	236 (4.0)
Diabetes	1986 (1.0)	32 (0.0)	188 (14.3)	1766 (29.9)
Adiposity	494 (0.2)	104 (0.1)	99 (7.5)	291 (4.9)
Down syndrome	1058 (0.5)	1041 (0.5)	13 (1.0)	4 (0.1)
Selected complications, *N* (%)				
RSV pneumonia, J12.1	65,215 (31.8)	60,972 (30.8)	793 (60.4)	3450 (58.5)
RSV bronchitis, J20.5	65,165 (31.7)	62,402 (31.5)	470 (35.8)	2293 (38.9)
RSV bronchiolitis, J21.0	79,336 (38.6)	79,080 (39.9)	63 (4.8)	193 (3.3)
RSV classified elsewhere, B97.4*	3401 (1.7)	2828 (1.4)	98 (7.5)	475 (8.1)
Viral pneumonia, not by RSV	908 (0.4)	825 (0.4)	29 (2.2)	54 (0.9)
Pneumonia, bacterial	4512 (2.2)	3953 (2.0)	138 (10.5)	421 (7.1)
Sepsis	546 (0.3)	272 (0.1)	58 (4.4)	216 (3.7)
Otitis media	6932 (3.4)	6901 (3.5)	18 (1.4)	13 (0.2)
ARDS	143 (0.1)	74 (0.1)	28 (2.1)	41 (0.7)
Febrile seizure	2158 (1.1)	2147 (1.1)	3 (0.2)	8 (0.1)
Treatment/fatality, *N* (%)				
Intensive care	5191 (2.5)	4346 (2.2)	205 (15.6)	640 (10.8)
Prophylactic isolation	65,377 (31.8)	61,579 (31.1)	590 (44.9)	3208 (54.4)
Extracorporeal blood circulation (ECMO)	47 (0.0)	26 (0.0)	12 (0.9)	9 (0.2)
Hospital stay in days, median (IQR)	5 (3;7)	4 (3;7)	7 (4;11)	8 (5;11)
In-hospital fatality	612 (0.3)	103 (0.1)	51 (3.9)	458 (7.8)

Data source: German Statistical Office

Data are *N* (percent) or median (quartiles), unless otherwise specified. Cases were assigned to study years by date of hospital discharge. Sex is unknown for 13 patients: they were included as female. ICD-10-GM codes are either primary or secondary diagnosis in RSV pneumonia, RSV bronchitis, RSV bronchiolitis, as multiple nominations of these RSV codes per patient are possible. The code B97.4 (RSV classified elsewhere) and all selected complications, risk factors/underlying chronic conditions, and treatments were reported solely as secondary diagnosis

*ARDS* Acute respiratory distress syndrome. *LRT* Lower respiratory tract

For all reported variables listed in this Table, the differences among age groups were highly significant (*p* < 0.001; Chi-Squared test) *In some of the patients with RSV as primary diagnosis, the RSV-code B97.4 was additionally reported as a secondary diagnosis

### Selected risk factors/underlying chronic conditions and complications

Overall, the most frequently reported selected risk factor/underlying chronic condition was circulatory system disease (3.6%), followed by chronic disease of the lower respiratory tract (LRT) (2.4%) and nervous system disorders (1.9%) (Table 1). Children < 18 years reported few pre-existing risk factors/underlying chronic conditions, with chronic diseases of the lower respiratory tract (LRT) and nervous system disorders having the highest proportions, 1.6% and 1.2% of 198,139 children, respectively. Among child age groups (Supplementary Table 2), those > 4 years old usually had higher rates of pre-existing conditions than younger children. The rate of pre-existing nervous system disease, circulatory system disorder and immune disorder increased with child age. Adults showed highest rates for circulatory system disease (42.0%), immune disorder (35.7%), chronic disease of the LRT (22.9%), and nervous system disorder (18.7%). In seniors, the highest proportions of risk factors/underlying chronic conditions were documented for circulatory system disease (82.9%), diabetes (29.9%), chronic disease of LRT (24.0%), nervous system disorders (21.8%) and immune disorder (14.3%).

RSV pneumonia and bronchitis were documented in 31.8 and 31.7% of all RSV-coded hospitalizations (Table 1). RSV bronchiolitis (38.6% of all) was most frequently documented among children < 1 year old (46.3%) (Supplementary Table 2). Bacterial pneumonia was reported as a complication in 2.0% of all children, 10.5% of adults, and 7.1% of seniors. Among the children, those 5–9 years old reported having bacterial pneumonia 6.3% of the time, 10–14 years old had it 9.5% of the time, and 15–17 years olds had it 6.6% of the time. Sepsis was documented in 0.1% of children, 4.4% of adults and 3.7% of seniors. Otitis media was documented in 4% of all children, with the highest rates in 1–4 year-olds (8.2%) and 5–9 year-olds (3.8%).

### Treatment and outcomes

Overall, 2.5% of all patients with RSV-coded hospitalizations required intensive care (2.2% of children, 15.6% of adults, 10.8% of seniors) (Table 1). Among children, intensive care treatment was reported for 2.1%, 2.2%, 9.6%, 14.2% and 16.2% of those who were < 1, 1–4, 5–9, 10–14, and 15–17 years old, respectively (Supplementary Table 2). Extracorporeal blood circulation (ECMO) was reported in 26 children (0.01%), 12 adults (0.9%) and 9 seniors (0.2%). The median length of hospital stay was 5 days for all age groups (IQR 3–7), with children reporting a median stay of 4 days (IQR 3; 7) and seniors reporting a median stay of 8 days (IQR 5; 11). Among children, those 1–4 years old reported a median hospital stay of 4 days (IQR 3; 6) and those 15–17 years old a median hospital stay of 5 days (IQR 3; 10). There were 612 in-hospital fatalities (0.3% of all), with 103 cases among children (0.1%), 51 among adults (3.9%) and 458 among seniors (7.8%). Additional stratification of RSV-related hospitalizations by ten age groups (Supplementary Table 3) showed a continuous increase of fatality rate with increasing age.

### Hospitalization incidence

During the 10-year observation period, overall annual hospitalization incidence estimates based on cases with ICD-10-coded RSV as primary diagnosis varied between a minimum value of 18 (2010/2011) and a maximum of 36.5 (2019) per 100,000 persons (Table 2). In children < 18 years, annual RSV-coded hospitalization incidence was between 110 (2011) and 199 (2019), in adults 18–59 years between 0.06 (2010) and 0.91 (2019), and in seniors > 59 years between 0.11 (2010) and 11.36 (2019). The hospitalization incidence in 2019 was 1.7 times higher than the incidence in 2010 among children, 15.1 times higher among adults, and 103.3 times higher among seniors. During the years 2017 to 2019, annual hospitalization incidences in adults (0.4–0.9 per 100,000) and in seniors (4.8–11.4 per 100,000) were higher than during the years 2010 to 2016 (adults < 0.23, seniors < 1.14).

**Table 2:** Annual RSV-coded hospitalizations (ICD-10 GM codes J12.1/J20.5/J21.0 as primary diagnosis; *n* = 205,352) per 100,000 persons in Germany between January 2010 and December 2019, stratified by age groups (< 18, 18–59, and > 59 years)

	2010	2011	2012	2013	2014	2015	2016	2017	2018	2019
All	18.44	18.07	23.04	23.48	21.26	23.44	26.16	30.05	26.07	36.53
< 18	112.60	109.68	141.03	168.26	130.95	142.12	157.60	172.28	149.39	199.34
18–59	0.06	0.12	0.11	0.18	0.09	0.20	0.22	0.54	0.43	0.91
> 59	0.11	0.20	0.18	0.53	0.24	1.02	1.13	5.68	4.84	11.36

Data source: German Statistical Office

Cases were assigned to study years by date of hospital discharge. Note that reported RSV-coded hospitalization incidences are minimum estimates based on RSV reported as primary ICD-10 diagnosis

### Direct hospitalization costs per patient

Total direct hospitalization cost for all patients in Germany with RSV infection coded as primary diagnosis from 2010 to 2019 was 704,014,781€. The median (IQR) per patient cost from 2010 to 2019 amounted to 2391€ (2310€; 3821€) and the mean (SD) per patient cost was 3429€ (± 4619€) (Table 3). Females had higher mean costs when compared to males. Hospitalized patients < 18 years had a mean (SD) per patient cost of 3335€ (± 4177€), adults 18–59 years had the highest per patient mean (SD) cost, 7215€ (± 13,564€), and seniors > 59 years had the second highest per patient mean (SD) cost, 5731€ (± 10,338€). Among children, those who were 10–14 years and 15–17 years reported the highest per patient mean (SD) costs, 5933€ (± 8316) and 6432€ (± 9743), respectively. The lowest per patient mean (SD) costs were observed in the 1–4 year old group, 3286€ (± 4594). Annual direct per patient hospitalization costs are given in Supplementary Table 4.

**Table 3:** Direct per patient hospitalization costs* of individuals with an RSV-coded hospitalization (ICD-10 GM codes J12.1/J20.5/J21.0 as primary diagnosis) in Germany, January 2010–December 2019

	*N* (%)	Median € (IQR)	Mean € (± SD)
All	205,352 (100)	2391 (2310; 3821)	3429 (± 4619)
Sex			
Female	90,466 (44.1)	2391 (2310; 3852)	3446 (± 4628)
Male	114,886 (55.9)	2391 (2309; 3786)	3415 (± 4611)
Age, years			
< 18	198,139 (96.5)	2391 (2309; 3768)	3335 (± 4177)
18–59	1313 (0.6)	3982 (2155; 8074)	7215 (± 13,564)
> 59	5900 (2.9)	3982 (2364; 4453)	5731 (± 10,338)
Age, years			
< 1	152,989 (77.2)	2389 (2313; 3709)	3329 (± 3994)
1–4	43,470 (21.9)	2690 (1788; 3977)	3286 (± 4594)
5–9	1248 (0.6)	3608 (1935; 4086)	4827 (± 6963)
10–14	296 (0.1)	3674 (1963; 4623)	5933 (± 8316)
15–17	136 (0.1)	3857 (1759; 5973)	6432 (± 9743)

Data source: German Statistical Office

^*^Standardized to 2019 EUR. Cases were assigned to study years by date of hospital discharge. Cost is unknown for 12 patients. Sex is unknown for 13 of 205,352 persons. These persons were included as female. For all reported variables listed in this Table, the differences among sex and age groups were highly significant (*p* < 0.001), using Mann–Whitney *U*-test or Kruskal–Wallis test, as appropriate). Total hospitalization cost for all patients was 704,014,781€

The per patient cost was assessed between those with selected risk factors/underlying chronic conditions, complications and treatment and those without, and an x-fold change named “∆” that notes how much higher or lower the cost of the affected vs. unaffected was calculated. The highest difference was observed in patients with and without an ECMO treatment, where those who required the treatment had on average 16.88 times higher cost than those without. Patients with an acute respiratory distress syndrome complication diagnosis had a 12.85 times higher cost than those without and those with sepsis as a complication had 7.27 times higher per patient cost than those without. Patients who required intensive care had an average per patient cost that 4.60 times higher than those who did not, and patients who needed CPAP treatment had per patient costs that were 4.66 times higher than patients who did not. For selected risk factors/underlying chronic conditions, most patients had between 1.7 and 2.6 times higher costs, with nervous system disorders having the highest x-fold difference (2.66 times higher) and bronchial asthma having the lowest (1.06 times higher) (Table 4).

**Table 4:** Direct per patient hospitalization costs* of individuals with an RSV-coded hospitalization (ICD-10 code for RSV as primary diagnosis) in Germany, January 2010–December 2019

	*N* (%)	Median € (IQR)	Mean (± SD)	X-fold ∆ between affected vs unaffected†
Selected risk factors/underlying chronic conditions				
Immune disorder	1907 (0.9)	4440 (2379; 9453)	8612 (± 17,242)	2.55 ( +)
Chronic disease of LRT (without asthma)	3283 (1.6)	3690 (2361; 4087)	5841 (± 12,414)	1.72 ( +)
Bronchial asthma	1614 (0.8)	3608 (2309; 3977)	3616 (± 4564)	1.06 ( +)
Disease of circulatory system	7484 (3.6)	3985 (2373; 7425)	8365 (± 17,484)	2.58 ( +)
Disease of nervous system	4000 (1.9)	3985 (2379; 7410)	8824 (± 17,593)	2.66 ( +)
*Cerebral palsy*	1014 (0.5)	3992 (2593; 7093)	8356 (± 15,278)	2.45 ( +)
Diabetes	1986 (1.0)	3985 (2379; 6018)	6552 (± 12,834)	1.93 ( +)
Adiposity	494 (0.2)	3978 (2361; 7544)	8281 (± 18,679)	2.42 ( +)
Cystic fibrosis	58 (0.0)	5910 (5095; 6496)	8913 (± 11,842)	2.60 ( +)
Down syndrome	1058 (0.5)	3992 (2602; 6378)	7722 (± 14,634)	2.27 ( +)
Prematurity-related disorders	391 (0.2)	4088 (2414; 9461)	8503 (± 10,160)	2.49 ( +)
Congenital malformation of circulatory system	2955 (1.4)	3707 (2379; 6290)	7665 (± 18,134)	2.28 ( +)
Congenital malformation of nervous system	777 (0.4)	3884 (2387; 6472)	7937 (± 14,281)	2.33 ( +)
Selected complications				
RSV pneumonia, J12.1	65,215 (31.8)	3978 (3676; 4098)	5059 (± 6936)	1.90 ( +)
RSV bronchitis, J20.5	65,165 (31.7)	2224 (2073; 2373)	2395 (± 1825)	0.61 ( – )
RSV bronchiolitis, J21.0	79,336 (38.6)	2381 (2325; 2487)	3025 (± 3431)	0.82 ( – )
RSV classified elsewhere, B97.4	3401 (1.7)	2434 (2333; 3989)	3870 (± 6675)	1.13 ( +)
Viral pneumonia, not by RSV	908 (0.4)	3992 (3637; 4890)	7395 (± 15,882)	2.17 ( +)
Pneumonia, bacterial	4512 (2.2)	3992 (3164; 6495)	8166 (± 15,843)	2.46 ( +)
Respiratory distress in newborns	1711 (0.8)	4035 (3633; 5729)	5556 (± 5764)	1.63 ( +)
Sepsis	546 (0.3)	10,846 (5373; 26,639)	24,529 (± 36,208)	7.27 ( +)
Otitis media	6932 (3,4)	2434 (2241,3852)	3064 (± 2435)	0.89 ( – )
ARDS	143 (0.1)	34,354 (17,827; 47,886)	43,681 (± 44,708)	12.85 ( +)
Febrile seizure	2158 (1.1)	2368 (1670; 3786)	3826 (± 7046)	1.12 ( +)
Treatment/outcome				
Intensive care	5191 (2.5)	8073 (3652; 22,549)	14,465 (± 21,502)	4.60 ( +)
Prophylactic isolation	65,377 (31.8)	2389 (2319; 3963)	3562 (± 5042)	1.06 ( +)
Extracorporeal blood circulation (ECMO)	47 (0.0)	45,105 (28,759; 88,883)	57,664 (± 41,607)	16.88 ( +)
CPAP	3334 (1.6)	8759 (7065; 24,030)	15,079 (± 16,951)	4.66 ( +)
In-hospital fatality	612 (0.3)	5621 (3010; 11,842)	14,076 (± 31,048)	4.14 ( +)

Data source: German Statistical Office

For all reported variables listed in this table, the differences between the per patient costs of those affected vs. unaffected were highly significant (*p* < 0.001), except for otitis media, where the *p*-value was 0.019 (using Mann–Whitney). Total hospitalization cost for all patients was 704,014,781€

*ARDS* Acute respiratory distress syndrome. *LRT* Lower respiratory tract. *CPAP* continuous positive airway pressure

^*^Standardized to 2019 EUR

^†^Calculated from mean cost values. Cases were assigned to study years by date of hospital discharge. Cost is unknown for 12 patients. ICD-10-GM codes are either primary or secondary diagnosis in RSV pneumonia, RSV bronchitis, RSV bronchiolitis; multiple nominations of these RSV codes per patient are possible. The code RSV classified elsewhere and all other selected complications, risk factors/underlying chronic conditions, and treatments were reported solely as secondary diagnosis

## Discussion

### Clinical burden of RSV-coded hospitalizations

The study analyzed clinical and direct medical cost data of 205,352 patients hospitalized with a primary ICD-10-coded diagnosis for RSV lower respiratory tract disease during the years 2010–2019 in Germany. The large majority were children < 4 years old, previously healthy and with low rates of severe complications and intensive care treatment. Children who were 5–17 years old were few, had higher proportions of risk factors/underlying chronic conditions and more often required intensive care.

Adults and seniors accounted only for 0.6% and 2.9%, respectively, of the total number of RSV-coded hospitalizations in the 10-year period, but had higher proportions of risk factors/underlying chronic conditions, complications, intensive care treatment, ECMO treatment, and in-hospital fatalities when compared to children. In fact, in-hospital fatality rate steadily increased with patient age, with 75% of all 612 reported fatalities observed in seniors. During study years 2017–2019, there was a sudden increase in the number of adults and seniors with an RSV-coded hospitalization, consequently we also observed a sudden increase in the number of fatal cases during this time period (data not shown). Previous studies had already shown that acute RSV LRTI in adults with significant underlying chronic conditions can lead to severe, often life-threatening, complications [22, 23]. Especially adults with comorbidities such as chronic respiratory disease, cardiovascular disease, and compromised immune systems have an increased susceptibility to severe RSV disease that requires hospitalizations [24–27]. A systemic literature review by Savic et al. [28] found the in-hospital fatality rate of RSV patients ≥ 60 years old in high-income countries to be 7.1%, which is similar to the presented in-hospital fatality rate of 7.8% in seniors > 59 years old in Germany.

An interesting observation on clinical treatment was that 31.1% of children with RSV-coded hospitalization were isolated as a prophylactic measure, in contrast to higher proportions in adults and seniors. We suspect that the isolated adults and seniors with RSV-coded hospitalizations often represented high-risk patients.

The overall number of adult and senior RSV-coded hospitalizations or RSV-associated pneumonia was low compared to patients with influenza-coded hospitalizations or influenza-associated pneumonia, which accounted for hospitalizations of 34,829 adult and 73,286 senior patients during the same 10-year observation period in Germany, including 6,885 and 24,235 patients with influenza-associated pneumonia [17].

### RSV-coded hospitalization incidence

In all study years, the hospitalization incidence based on RSV-coding was considerably higher among children (in 2019, 219.1 times higher than adults and 17.5 times higher than seniors). While hospitalization incidences were below 0.2/100,000 in adults and below 1.1/100,000 in seniors from 2010 to 2015, a conspicuous increase was observed from 2016 to 2017 (2.5-fold in adults, 5.0-fold in seniors) and a further increase from 2018 to 2019 (2.1-fold in adults, 2.3-fold in seniors). This increase was not observed in the children population. Hence, it may be more likely attributed to an increasing awareness of RSV as a possible cause of severe disease among adults and seniors and, consequently, an increasing use of virological testing rather than to a change in virus characteristics. Rapid molecular testing for RSV seems to contribute to better patient outcomes in older adults, however, it is not widely implemented in healthcare settings [29]. Seniors are known to be inconsistently tested for RSV in hospitals, meaning the knowledge on the impact of the true effects of the disease is incomplete [29]. Adults commonly have low viral titers and an overall shorter duration of viral shedding compared to children, which may limit RSV detection and diagnosis [30]. Since our study specifically focuses on patients with an ICD-10-coded RSV primary diagnosis usually based on laboratory confirmation, the difficulty associated with testing adults can definitely impact the number of adults and seniors included in our analysis.

The hospitalization incidence may also be affected by our case definition, selecting the well-defined group of patients with an RSV-code as primary diagnosis. Hence, in addition to this main analysis group (‘Validated’ cases), we additionally performed an analysis on two further patient groups with broader inclusion criteria: i) a group that included RSV infections as primary diagnosis plus RSV infections listed as any secondary diagnosis as long as the primary diagnosis was a “J” ICD-10-GM code indicating a disease of the respiratory system (‘Searched’ cases), and ii) a group that included all patients that had either a primary or a secondary diagnosis of RSV (‘Reported’ cases). The results of these additional analysis revealed that there was little difference in the age distribution among the three analysis groups (Supplementary Table 5). The overall number of patients included was similar among the Validated, Searched, and Reported groups (205,352 vs. 214,629 vs. 228,212), and the distributions of selected risk factors/underlying chronic conditions, complications, and treatments were also similar. Children were still the large majority in all analysis groups. It is important to note, however, that the ‘Reported’ group had a higher proportion of adults and seniors—almost double compared to the ‘Validated’ group. This is most likely the case because ‘Reported’ cases included approximately 12,500 patients with RSV as a secondary diagnosis and a constantly higher rate for patients with selected risk factors/underlying chronic conditions compared to the ‘Validated’ group. This also translates to the overall in-hospital fatality rate, essentially doubling in the Reported group, with the majority of fatalities again in the senior age group.

### Costs of RSV-coded hospitalizations

The cost analysis quantifies the direct healthcare costs of RSV-confirmed hospitalizations in Germany from January 2010 to December 2019. In the 10 years included in the study, 205,352 hospitalized patients with an ICD-10-coded primary RSV infection (J12.1/J20.5/J21.0 primary diagnosis) amounted an estimated 704,014,781€ in just direct costs. The highest mean per patient costs were reported from 2017 to 2019; however, the higher costs do not reflect higher costs due to a more serious course of disease or due to a higher number of elderly patients with RSV during these years.

In a similar study conducted by our research group looking at the per-patient cost of influenza-coded hospitalizations in Germany, the mean cost for all influenza patients over the same 10-year span was 3521€, which is higher (by 92€) to that of RSV-related hospitalizations in our study [17]. The median per-patient cost for influenza-coded hospitalizations is about 75% of that of RSV-coded hospitalizations (1805€ vs. 2391€), which may be due to the fact that the ICD-10-GM codes for influenza available as primary codes are not restricted only to patients with LRTI but may also cover less severe manifestations of the disease.

In the present study, adults and seniors had the highest per-patient costs. In fact, the mean (± SD) per-patient cost for all patients hospitalized due to an RSV infection peaks among seniors 60–69 years old: 8442€ (± 18,404€) (Supplementary Table 3). Other studies on RSV burden have concluded that driving factors of increased costs are older age and the presence of risk factors/underlying chronic conditions [31]. Similarly, in our previous influenza study, patients 60–69 years old also reported the highest per-patient cost.

Our analysis also shows that children 10–17 (who are usually not considered when discussing RSV) had higher per-patient costs than the other children age groups, probably due to their higher rate of risk factors/underlying chronic conditions for a severe course of disease. Children 15–17 years old had a median per-patient cost of 3857€ while children < 1 year old reported a median per-patient cost which is similar to the overall median per-patient cost of 2391€. Mean costs per-patient followed a similar trend. In fact, the median and mean costs of children 10–14 and 15–17 years old were more similar to that of adults 18–59 and > 59 years old than that of younger children.

When comparing the difference in cost between the risk factors/underlying chronic conditions of patients hospitalized due to an RSV infection, it is obvious that all selected risk factors/underlying chronic conditions in our study increased the total cost for each patient. Adults and seniors had the highest rates of immune disorders as well as the highest rates of sepsis, ARDS, intensive care and ECMO treatment, all factors with high impact on costs. A risk factor/underlying chronic condition that had a high x-fold change between those affected and those unaffected in children was prematurity-related disorders (2.49-fold increase). This emphasizes the fact that premature infants are particularly vulnerable to RSV infections and require more costly care.

### Limitations

There are some relevant limitations to the interpretation of our study results. RSV diagnostic testing is not routinely performed in adult and senior patients upon presenting at the hospital with respiratory symptoms, most likely due to the lack of specific antiviral therapy [29]. In addition, since DeStatis only reports costs reimbursed by the German sickness fund, out-of-pocket payments are not calculated in the costs reported by our analysis. Our study has particularly great external validity since the entire German population is included, but there is low internal validity due to potential coding issues—especially since all hospitals in Germany are included [17]. ICD-10-GM codes chosen (J12.1, J20.5, J21.0) were specific for patients who had either tested positive for RSV or were classified as RSV patients due to the epidemiological situation and had received a primary diagnosis of an RSV infection. However, this method does not capture hospitalized patients who might have had an RSV infection but were not tested or RSV disease was not suspected from the epidemiological situation. If solely the primary diagnosis is used to identify patients with RSV-coded hospitalization (Validated’ cases), the true number may be underestimated, as shown previously on the burden of influenza-associated hospitalizations [17, 32]. In contrast, including all patients with any primary or secondary diagnoses of RSV (‘Reported’ cases) is likely to overestimate the true hospitalization incidence, as this group may include patients with an RSV-unrelated primary diagnosis and ‘incidental’ RSV infection. Therefore, it is evident that our main analysis represents only a minimum estimate of the true burden of disease of RSV hospitalizations.

### Conclusion

Our 10-year retrospective analysis of RSV-related hospitalizations showed German inpatient clinical data and associated direct per-patient medical costs based on ICD-10-GM codes. The economic burden of RSV-related hospitalizations in Germany is substantial, even when only considering cases where an RSV LRTI is reported as the primary discharge diagnosis. Children have been traditionally targeted in RSV awareness efforts, as they represent the vast majority of RSV cases. However, adults and seniors hospitalized for RSV are at a higher risk of severe complications, require more costly treatments, have a higher fatality rate, and their true number is likely to be underestimated due to lack of routine testing. The associated mortality, morbidity, and healthcare costs might be decreased with the development and approval of new prophylactic treatments and vaccines, which ideally should also target adults and seniors in addition to children.

## Supplementary Information

Below is the link to the electronic supplementary material.

Supplementary file1 (DOCX 37 kb)

## Data Availability

Data Source: Research Data Center (RDC) of the Federal Statistical Office and Statistical Offices of the Länder (Germany), DRG-Statistik 2010–2019, doi 10.21242/23141.2010.00.00.1.1.0 to 10.21242/23141.2019.00.00.1.1.0; own calculations (project 4458-2021). The analysis programs and the resultant data extractions are not publicly available.

## Abbreviations

ARDS: Acute respiratory distress syndrome
CNS: Central nervous system
DeStatis: Federal Statistical Office of Germany (Deutsches Statistisches Bundesamt)
ECMO: Extracorporeal membrane oxygenation
HIV: Human Immunodeficiency Virus
ICD-10-GM: 10Th revision of the International Statistical Classification of Disease and Related Health Problems, German Modification
LRT: Lower respiratory tract
LRTI: Lower respiratory tract infection
OPS: Operational and Procedure Codes
RSV: Respiratory syncytial virus
