# Correction: Hospitalizations due to respiratory syncytial virus (RSV) infections in Germany: a nationwide clinical and direct cost data analysis (2010–2019)

**DOI:** 10.1007/s15010-024-02191-3

**Published:** 2024-03-25

**Authors:** Patricia Niekler, David Goettler, Johannes G. Liese, Andrea Streng

**Affiliations:** Department of Pediatrics, University Hospital of Würzburg, Josef-Schneider-Str. 2, 97080 Würzburg, Germany

**Correction: Infection** 10.1007/s15010-023-02122-8

This corrects the article “Hospitalizations due to respiratory syncytial virus (RSV) infections in Germany: a nationwide clinical and direct cost data analysis (2010–2019)” published online on 16 November 2023 by Niekler et al. in: Infection. 2023 Nov 16. 10.1007/s15010-023-02122-8. Epub ahead of print. PMID: 37973718.

In “Hospitalizations due to respiratory syncytial virus (RSV) infections in Germany: a nationwide clinical and direct cost data analysis (2010–2019)” by Niekler et al. [10.1007/s15010-023-02122-8], an error had occurred during the pre-selection of data (needed for technical reasons) on hospitalized patients with RSV at the German Statistical Office. Due to a typing error in the pre-selection program, the non-RSV ICD-10-GM code J12.0 (adenovirus pneumonia) had been used for pre-selection of datasets instead of the correct ICD-10 code J21.0 (RSV bronchiolitis). Therefore, a relevant number of children with RSV bronchiolitis as primary diagnosis had not been captured by our analysis programs running on the pre-selected dataset.

This has been corrected in the present article version: 10.1007/s15010-023-02122-8.

The authors sincerely regret this error.

