# Supplementary material for: Hospitalizations due to respiratory syncytial virus (RSV) infections in Germany: a nationwide clinical and direct cost data analysis (2010–2019)
Source: Infection. 2023 Nov 16;52(5):1715–24. doi: 10.1007/s15010-023-02122-8 (PMC11499329; doi:10.1007/s15010-023-02122-8)
Supplement: Supplementary file 1 — Supplementary file1 (DOCX 37 kb) [file 15010_2023_2122_MOESM1_ESM.docx]

| **Supplementary Table 1** Relevant, pre-selected ICD-10-GM and Operation and Procedure (OPS) codes with their respective characterization | |
| --- | --- |
| **ICD-10-GM** **Code** | **Categorization** |
| C00-99, B20-24, D80-90, Z94 | Immunocompromised state (malignant neoplasms, HIV, organ transplant,etc) |
| J40-J44, J47 | Chronic illness of lower respiratory tract |
| J45-J46 | Bronchial asthma |
| I00-I99 | Diseases of circulatory system |
| G00-G99 | Diseases of the nervous system |
| G80-G83 | Cerebral palsy |
| E10-E14 | Diabetes |
| E66 | Adiposity |
| Q90 | Down syndrome |
| O00-99 | Pregnancy |
| P07 | Disorders associated with prematurity and low birth weight |
| Q20-Q28 | Congenital malformation of circulatory system |
| Q00-Q07 | Congenital malformation of nervous system |
| J12.1 | RSV Pneumonia |
| J20.5 | RSV Bronchitis |
| J21.0 | RSV Bronchiolitis |
| B97.4! | RSV as the cause of diseases classified elsewhere |
| J10.0, J11.0, J12.0, J12.2, J12.3, J12.8, J12.9 | All viral pneumonia, not caused by RSV |
| J13-J18 | Bacterial Pneumonia |
| P22 | Respiratory distress in newborns |
| A40-A41 | Sepsis |
| H65.0, H65.1, H65.9, H66.0, H66.4, H66.9, H67.1 | Otitis Media |
| J80 | ARDS |
| R56 | Febrile Seizure |
| Z290 | Prophylactic isolation |
| **OPS-Code** | **Selected treatment codes** |
| 8-980 **or** 8-98d **or** 8-98f | Intensive care treatment |
| 8-852 | Extracorporeal blood circulation |
| 8-7110, 8-7120 | CPAP |
| These codes were used to define groups and subgroups for the present analyses on RSV-associated hospitalizations, derived from the database on German Hospital Statistics from the Research Data Centers of the German Federal Statistical Office, Germany, 2010-2019. RSV: Respiratory Syncytial Virus. ARDS: Acute respiratory distress syndrome. HIV: Human Immunodeficiency Virus. CPAP: continuous positive airway pressure treatment. | |

**Supplementary Results**

| **Supplementary Table 2** General characteristics of children with RSV-coded hospitalization (ICD-10-GM RSV code J12.1, J20.5 or J21.0 reported as primary diagnosis) in Germany, January 2010-December 2019, stratified by five age groups (<1, 1-4, 5-9, 10-14, 15-17 years). | | | | | |
| --- | --- | --- | --- | --- | --- |
|  | Age, years | | | | |
|  | <1 | 1-4 | 5-9 | 10-14 | 15-17 |
| All cases, N (%) | 152,989 (100) | 43,470 (100) | 1,248 (100) | 296 (100) | 136 (100) |
|  |  |  |  |  |  |
| Age (years), mean (±SD) | 0 (±0) | 1.50 (±0.79) | 6.16 (±1.32) | 11.72 (±1.37) | 15.87 (±0.79) |
| Female sex, N(%) | 66,185 (43.3) | 19,600 (45.1) | 576 (46.2) | 124 (41.9) | 61 (44.9) |
| **Selected risk factors/underlying chronic conditions, N (%)** | | | | | |
| Immune disorder | 152 (0.1) | 311 (0.7) | 78 (6.3) | 30 (10.1) | 26 (19.1) |
| Chronic disease of LRT (without asthma) | 762 (0.5) | 922 (2.1) | 117 (9.4) | 28 (9.5) | 12 (8.8) |
| Bronchial asthma | 502 (0.3) | 703 (1.6) | 105 (8.4) | 22 (7.4) | 9 (6.6) |
| Disease of circulatory system | 1,253 (0.8) | 662 (1.5) | 68 (5.4) | 26 (8.8) | 29 (21.3) |
| Disease of nervous system | 709 (0.5) | 1,289 (3.0) | 294 (23.6) | 122 (41.2) | 54 (39.7) |
| *Cerebral palsy* | 64 (0.0) | 386 (0.9) | 157 (12.6) | 71 (24.0) | 38 (27.9) |
| Down syndrome | 512 (0.3) | 492 (1.1) | 28 (2.2) | 6 (2.0) | 3 (2.2) |
| Prematurity-related disorders | 355 (0.2) | 36 (0.1) | 0 (0.0) | 0 (0.0) | 0 (0.0) |
| Congenital malformation of circulatory system | 2,268 (1.5) | 622 (1.4) | 39 (3.1) | 11 (3.7) | 5 (3.7) |
| Congenital malformation of nervous system | 374 (0.2) | 318 (0.7) | 58 (4.6) | 22 (7.4) | 4 (2.9) |
| **Selected complications, N (%)** | | | | | |
| RSV pneumonia | 37,674 (24.6) | 22,319 (51.3) | 731 (58.6) | 174 (58.8) | 74 (54.4) |
| RSV bronchitis | 47,794 (31.2) | 14,027 (32.3) | 421 (33.7) | 104 (35.1) | 56 (41.2) |
| RSV bronchiolitis | 70,890 (46.3) | 8,046 (18.5) | 111 (8.9) | 25 (8.4) | 8 (5.9) |
| RSV classified elsewhere | 2,191 (1.4) | 602 (1.4) | 26 (2.1) | 6 (2.0) | 3 (2.2) |
| Viral pneumonia, not by RSV | 278 (0.2) | 509 (1.2) | 29 (2.3) | 3 (1.0) | 6 (4.4) |
| Pneumonia, bacterial | 2,290 (1.5) | 1,548 (3.6) | 78 (6.3) | 28 (9.5) | 9 (6.6) |
| Sepsis | 200 (0.1) | 58 (0.1) | 10 (0.8) | XXX | XXX |
| Otitis Media | 3,277 (2.1) | 3,569 (8.2) | 47 (3.8) | 4 (1.4) | 4 (2.9) |
| Febrile Seizure | 579 (0.4) | 1,532 (3.5) | 29 (2.3) | XXX | XXX |
| **Treatment/Fatality, N (%)** | | | | | |
| Intensive care | 3,194 (2.1) | 968 (2.2) | 120 (9.6) | 42 (14.2) | 22 (16.2) |
| Prophylactic isolation | 47,038 (30.7) | 13,989 (32.2) | 421 (33.7) | 91 (30.7) | 40 (29.4) |
| Extracorporeal blood circulation (ECMO) | 18 (0.0) | 6 (0.0) | XXX | 0 (0) | XXX |
| CPAP | 3,073 (2.0) | 220 (0.5) | 25 (2.0) | 9 (3.0) | 6 (4.4) |
| Hospital stay in day, median (IQR) | 5 (3;7) | 4 (3;6) | 4 (3;7) | 5 (3;9) | 5 (3;10) |
| In-hospital fatality | 38 (0.0) | 54 (0.1) | 6 (0.5) | XXX | XXX |
| Data source: German Statistical Office.  Data are N (percent) or median (quartiles), unless otherwise specified. Cases were assigned to study years by date of hospital discharge. ICD-10-GM codes are either primary or secondary diagnosis in RSV pneumonia, RSV bronchitis, RSV bronchiolitis, as multiple nominations of these RSV codes per patient are possible. The code B97.4 (RSV classified elsewhere) and all selected complications, risk factors/underlying chronic conditions, and treatments were reported solely as secondary diagnosis. LRT: Lower respiratory tract. For all reported variables listed in this Table, the differences among age groups were highly significant (p<0.001; Chi-Squared test), except for ‘RSV classified elsewhere’ (p=0.219). If at least one age group contained less than 3 patients, data was removed for patient data protection (XXX). | | | | | |

| **Supplementary Table 3** General characteristics of patients with RSV-coded hospitalization (ICD-10-GM RSV code J12.1, J20.5 or J21.0 reported as primary diagnosis) in Germany, January 2010-December 2019, stratified by ten age groups (<10, 10-19, 20-29, 30-39, 40-49, 50-59, 60-69, 70-79, 80-89, ≥90 years). | | | | | | | | | | |
| --- | --- | --- | --- | --- | --- | --- | --- | --- | --- | --- |
|  | Age, years | | | | | | | | | |
|  | <10 | 10-19 | 20-29 | 30-39 | 40-49 | 50-59 | 60-69 | 70-79 | 80-89 | ≥90 |
| All cases, N (%) | 197,707 (100) | 474 (100) | 146 (100) | 186 (100) | 240 (100) | 699 (100) | 1,068 (100) | 1,937 (100) | 2,232 (100) | 663 (100) |
|  |  |  |  |  |  |  |  |  |  |  |
| Age (years), mean (±SD) | 0.37 (±0.9) | 13.51 (±2.7) | 24.56 (±3.0) | 34.43 (±2.8) | 45.12 (±2.9) | 55.17 (±2.8) | 64.72 (±2.9) | 75.27 (±2.9) | 84.06 (±2.8) | 92.77 (±2.5) |
| Female sex, N (%) | 86,361 (43.7) | 202 (42.6) | 67 (45.9) | 96 (51.6) | 117 (48.8) | 304 (43.5) | 526 (49.3) | 966 (49.9) | 1,349 (60.4) | 478 (72.1) |
| **Selected risk factors/underlying chronic conditions, N (%)** | | | | | | | | | | |
| Immune disorder | 541 (0.3) | 67 (14.1) | 40 (27.4) | 54 (29.0) | 96 (40.0) | 268 (38.3) | 342 (32.0) | 322 (16.6) | 160 (7.2) | 17 (2.6) |
| Chronic disease of LRT (without asthma) | 1,801 (0.9) | 42 (8.9) | 15 (10.3) | 15 (8.1) | 41 (17.1) | 159 (22.7) | 283 (26.5) | 457 (23.6) | 394 (17.7) | 76 (11.5) |
| Disease of circulatory system | 1,983 (1.0) | 65 (13.7) | 27 (18.5) | 37 (19.9) | 95 (39.6) | 383 (54.8) | 733 (68.6) | 1,607 (83.0) | 1,968 (88.2) | 586 (88.4) |
| **Selected complications, N (%)** | | | | | | | | | | |
| Pneumonia, bacterial | 3,916 (2.0) | 45 (9.5) | 15 (10.3) | 14 (7.5) | 24 (10.0) | 77 (11.0) | 116 (10.9) | 132 (6.8) | 145 (6.5) | 28 (4.2) |
| Viral pneumonia, not by RSV | 816 (0.4) | 10 (2.1) | 4 (2.7) | 5 (2.7) | 5 (2.1) | 14 (2.0) | 13 (1.2) | 18 (0.9) | 20 (0.9) | 3 (0.5) |
| Sepsis | 268 (0.1) | 6 (1.3) | 6 (4.1) | 7 (3.8) | 7 (2.9) | 36 (5.2) | 68 (6.4) | 87 (4.5) | 53 (2.4) | 8 (1.2) |
| **Treatment/Fatality, N (%)** | | | | | | | | | | |
| Intensive care | 4,282 (2.2) | 72 (15.2) | 19 (13.0) | 21 (11.3) | 33 (13.8) | 124 (17.7) | 187 (17.5) | 245 (12.6) | 184 (8.2) | 24 (3.6) |
| Extracorporeal blood circulation | 25 (0.0) | 3 (0.6) | XXX | XXX | 0 (0.0) | 8 (1.1) | 7 (0.7) | XXX | 0 (0.0) | 0 (0.0) |
| Hospital stay in day, median (IQR) | 4 (3;7) | 5 (3;9) | 6 (3;10) | 5 (3;8) | 7 (3;9) | 7 (4;12) | 8 (5;12) | 8 (5;11) | 8 (6;11) | 8 (5;11) |
| In-hospital fatality | 98 (0.0) | 7 (1.5) | 3 (2.1) | 4 (2.2) | 9 (3.8) | 33 (4.7) | 52 (4.9) | 127 (6.6) | 184 (8.2) | 95 (14.3) |
| **Study year, N (%)** | | | | | | | | | | |
| 2010 | 15,001 (7.6) | 24 (5.1) | XXX | 9 (4.8) | 10 (4.2) | XXX | 8 (0.7) | 7 (0.4) | 8 (0.4) | XXX |
| 2011 | 14,388 (7.3) | 30 (6.3) | 9 (6.2) | 12 (6.5) | 8 (3.3) | 22 (3.1) | 20 (1.9) | 16 (0.8) | XXX | XXX |
| 2012 | 18,435 (9.3) | 27 (5.7) | 7 (4.8) | 9 (4.8) | 11 (4.6) | 22 (3.1) | 17 (1.6) | 14 (0.7) | XXX | XXX |
| 2013 | 21,937 (11.1) | 69 (14.6) | XXX | 10 (5.4) | 17 (7.1) | 42 (6.0) | 39 (3.7) | 48 (2.5) | 21 (0.9) | XXX |
| 2014 | 17,149 (8.7) | 23 (4.9) | XXX | 6 (3.2) | 9 (3.8) | 19 (2.7) | 21 (2.0) | 19 (1.0) | 12 (0.5) | XXX |
| 2015 | 18,899 (9.6) | 45 (9.5) | 12 (8.2) | 7 (3.8) | 20 (8.3) | 47 (6.7) | 75 (7.0) | 81 (4.2) | 59 (2.6) | 14 (2.1) |
| 2016 | 21,195 (10.7) | 36 (7.6) | 15 (10.3) | 18 (9.7) | 15 (6.3) | 50 (7.2) | 69 (6.5) | 81 (4.2) | 82 (3.7) | 26 (3.9) |
| 2017 | 23,257 (11.8) | 72 (15.2) | 33 (22.6) | 29 (15.6) | 46 (19.2) | 134 (19.2) | 213 (19.9) | 429 (22.1) | 517 (23.2) | 152 (22.9) |
| 2018 | 20,264 (10.2) | 53 (11.2) | 21 (14.4) | 31 (16.7) | 39 (16.3) | 105 (15.0) | 207 (19.4) | 379 (19.6) | 429 (19.2) | 116 (17.5) |
| 2019 | 27,182 (13.7) | 95 (20.0) | 33 (22.6) | 55 (29.6) | 65 (27.1) | 252 (36.1) | 399 (37.4) | 863 (44.6) | 1,092 (48.9) | 343 (51.7) |
| **Cost*** | | | | | | | | | | |
| Median (IQR) | 2,391 (2,309;3,768) | 3,756 (1,873;5,988) | 3,735 (1,622;6,538) | 3,029 (1,686;4,441) | 3,861 (1,686;7,845) | 3,986 (2,361;9,231) | 3,986 (2,364;8,114) | 3,982 (2,364;4,454) | 3,981 (2,363;4,440) | 3,981 (2,364;4,436) |
| Mean (±SD) | 3,329 (±4,160) | 6,147 (±8,691) | 7,228 (±16,281) | 4,439 (±5,622) | 6,972 (±16,409) | 8,063 (±13,558) | 8,442 (±18,404) | 6,037 (±9,800) | 4,684 (±5,516) | 4,003 (±2,285) |
| Data source: German Statistical Office.  Data are N (percent) or median (quartiles), unless otherwise specified. Cases were assigned to study years (January 2010 – December 2019) by date of hospital. ICD-10-GM codes are either primary or secondary diagnosis in RSV pneumonia, RSV bronchitis, RSV bronchiolitis, as multiple nominations of these RSV codes per patient are possible. The code B97.4 (RSV classified elsewhere) and all selected complications, risk factors/underlying chronic conditions, and treatments were reported solely as secondary diagnosis. LRT: Lower respiratory tract. For all reported variables listed in this Table, the differences among age groups were highly significant (p<0.001; Chi-Squared test). If at least one age group contained less than 3 patients, data was removed for patient data protection (XXX).*standardized to 2019 EUR. Data are N (percent) or median (quartiles), unless otherwise specified. Cases were assigned to study years (January 2010 – December 2019) by date of hospital discharge. Cost is unknown for 12 patients. | | | | | | | | | | |

| **Supplementary Table 4** Annual direct per-patient hospitalization costs* of patients with RSV-coded hospitalization (ICD-10-GM RSV code J12.1, J20.5 or J21.0 reported as primary diagnosis) in Germany, January 2010 - December 2019. | | | | |
| --- | --- | --- | --- | --- |
|  | N (%) | Median € (IQR) | Mean € (±SD) | (95% CI) † |
| **All** | 205,352 (100) | 2,391 (2,310;3,821) | 3,429 (±4,619) | (3409-3449) |
| **Study year** |  |  |  |  |
| 2010 | 15,076 (7.3) | 2,487 (2,278;3,978) | 3,397 (±3,648) | (3339-3455) |
| 2011 | 14,512 (7.1) | 2,606 (2,134;3,884) | 3,476 (±3,939) | (3412-3540) |
| 2012 | 18,549 (9.0) | 2,302 (2,147;3,667) | 3,219 (±3,020) | (3176-3262) |
| 2013 | 22,198 (10.8) | 2,345 (2,210;3,786) | 3,438 (±4,096) | (3384-3492) |
| 2014 | 17,266 (8.4) | 2,411 (2,186;3,608) | 3,169 (±3,590) | (3115-3223) |
| 2015 | 19,259 (9.4) | 2,389 (2,249;3,635) | 3,343 (±4,704) | (3277-3409) |
| 2016 | 21,587 (10.5) | 2,391 (2,319;3,985) | 3,280 (±3,883) | (3228-3332) |
| 2017 | 24,882 (12.1) | 2,379 (2,310;4,086) | 3,621 (±6,322) | (3542-3700) |
| 2018 | 21,644 (10.5) | 2,340 (2,330;4,441) | 3,635 (±5,693) | (3559-3711) |
| 2019 | 30,379 (14.8) | 2,370 (2,363;3,981) | 3,545 (±4,930) | (3490-3600) |
| *standardized to 2019 EUR. †of mean cost. Cases were assigned to study years (January - December) by date of hospital discharge. Cost is unknown for 12 patients. Total hospitalization cost for all patients was 704,014,781€ | | | | |

| **Supplementary Table 5** Additional analysis using three levels of inclusion criteria (‘Validated’, ‘Searched’, and ‘Reported’) for patients hospitalized with an RSV-coded infection in Germany, 2010-2019 | | | |
| --- | --- | --- | --- |
|  | **Validated^1^** | **Searched^2^** | **Reported^3^** |
| All | 205,352 | 214,629 | 228,212 |
| **Age groups, years** | | |  |
| < 18 | 198,139 (96.5) | 204,388 (95.2) | 213,016 (93.3) |
| 18-59 | 1,313 (0.6) | 1,970 (0.9) | 3,500 (1.5) |
| >59 | 5,900 (2.9) | 8,271 (3.9) | 11,696 (5.1) |
| **RSV codes** |  |  |  |
| RSV pneumonia | 65,215 (31.8) | 66,445 (31.0) | 69,902 (30.6) |
| RSV bronchitis | 65,165 (31.7) | 66,701 (31.1) | 71,263 (31.2) |
| RSV bronchiolitis | 79,336 (38.6) | 80,496 (37.5) | 83,112 (36.4) |
| RSV, classified elsewhere | 3,401 (1.7) | 8,993 (4.2) | 12,531 (5.5) |
| **Selected risk factors/underlying chronic conditions** | | |  |
| Immune disorder | 1,907 (0.9) | 2,464 (1.1) | 5,369 (2.4) |
| Chronic disease of LRT (without asthma) | 3,283 (1.6) | 5,298 (2.5) | 6,071 (2.7) |
| Disease of circulatory disease | 7,484 (3.6) | 10,032 (4.7) | 14,889 (6.5) |
| Disease of nervous system | 4,000 (1.9) | 4,888 (2.3) | 7,185 (3.1) |
| Diabetes | 1,986 (1.0) | 2,745 (1.3) | 4,111 (1.8) |
| **Selected complications** | | |  |
| Pneumonia, bacterial | 4,512 (2.2) | 6,880 (3.2) | 8,290 (3.6) |
| Viral pneumonia, not by RSV | 908 (0.4) | 1,366 (0.6) | 1,580 (0.7) |
| Otitis media | 6,932 (3.4) | 7,242 (3.4) | 7,611 (3.3) |
| Sepsis | 546 (0.3) | 784 (0.4) | 2,424 (1.1) |
| ARDS | 143 (0.1) | 317 (0.1) | 519 (0.2) |
| **Treatments/Outcomes** | | |  |
| Intensive care | 5,191 (2.5) | 6,232 (2.9) | 8,358 (3.7) |
| Extracorporeal blood circulation | 47 (0.0) | 150 (0.1) | 228 (0.1) |
| Fatality | 612 (0.3) | 928 (0.4) | 1,732 (0.8) |
| **Cost*** | | |  |
| Mean (±SD) | 3,429 (±4,619) | 3,555 (±6,147) | 4,477 (±12,255) |
| Median (IQR) | 2,391 (2,310;3,821) | 2,391 (2,308;3,786) | 2,414 (2,309;3,887) |
| Data source: German Statistical Office.  *standardized to 2019 EUR. Data are N (percent) or median (quartiles), unless otherwise specified. Cases were assigned to study years (January 2010 – December 2019) by date of hospital discharge. Cost is unknown for 12/15/128 patients in Validated/Searched/Reported. ICD-10-GM codes are either primary or secondary diagnosis; multiple nominations of RSV codes per patient are possible. The code ‘RSV classified elsewhere’ was reported solely as secondary diagnosis. LRT: Lower respiratory tract.  **^1^** ICD-10-coded RSV infection as primary diagnosis (J12.1, J20.5, J21.0)  **^2^** ICD-10-coded RSV infection as primary diagnosis AND RSV infection listed as any secondary diagnosis as long as the primary diagnosis is a “J” ICD-10 code (indicating a disease of the respiratory system)  ^3^all patients that had either a primary or secondary diagnosis of RSV | | | |
